
JOURNAL INFORMATION
==============================
NLM Title Abbreviation: Wirtschaftsdienst
Journal ID: Wirtschaftsdienst
NLM Journal ID: 101086612

Wirtschaftsdienst (Hamburg, Germany : 1949)

ISSN: 0043-6275

ARTICLE INFORMATION
==============================
PMCID: PMC7765690
PMID: 33390619
DOI: 10.1007/s10273-020-2792-2
Article ID: 2792
Article version: 1
Subjects: Kurz Kommentiert

Corona-Krise: Wer lässt sich impfen?

Corona crisis: who gets vaccinated?

Mayrhofer Thomas thomas.mayrhofer@hochschule-stralsund.de
1
Schmitz Hendrik hendrik.schmitz@uni-paderborn.de
2
1 grid.454249.a 0000 0001 0739 2463 Gesundheitsökonomie, VWL, Hochschule Stralsund, Zur Schwedenschanze 15, 18435 Stralsund, Deutschland
2 grid.5659.f 0000 0001 0940 2872 Fakultät für Wirtschaftswissenschaften, Universität Paderborn, Warburger Str. 100, 33098 Paderborn, Deutschland
Electronic publication date: 2020 Dec 26
Print publication date: 2020
Volume: 100
Issue: 12
First page: 905
Last page: 905
Copyright: © Der/die Autor(en) 2020
License: Dieser Artikel wird unter der Creative Commons Namensnennung 4.0 International Lizenz (https://creativecommons.org/licenses/by/4.0/deed.de) veröffentlicht.
Open Access wird durch die ZBW — Leibniz-Informationszentrum Wirtschaft gefördert.
License URL: https://creativecommons.org/licenses/by/4.0/

issue-copyright-statement: © The Author(s) 2020

==============================
DOI: 10.1007/s10273-020-2792-2

Corona-Krise

### Wer lässt sich impfen?

Bis Ende November hat COVID-19 (SARS-CoV-2) weltweit zu mehr als 60 Mio. bestätigten Infizierten und circa 1,5 Mio. bestätigten Todesfällen geführt, wobei davon ausgegangen wird, dass die Zahl der tatsächlichen Infektions- und Todesfälle deutlich höher liegt. Daher wurde in einer beispiellosen konzertierten Aktion die Entwicklung von Impfstoffen weltweit vorangetrieben. Bereits kurz nach dem Auftreten der ersten bekannten Fälle wurde im Januar 2020 das Genom des Virus SARS-CoV-2 sequenziert und daraufhin in verschiedenen Forschungsgruppen mit der Entwicklung eines Impfstoffs begonnen. Nur kurze Zeit später starteten im März 2020 bereits die ersten klinischen Studien am Menschen. Derzeit werden bereits mehr als 50 Impfstoffe am Menschen und eine Vielzahl mehr in vorklinischen Studien getestet. Die Geschwindigkeit dieses Prozesses ist bislang einmalig und zum großen Teil darauf zurückzuführen, dass verschiedene Regierungen schon vorab für Impfdosen bezahlt haben. Allein die USA haben im Rahmen der Operation Warp Speed bislang mehr als 10 Mrd. US-$ für die Entwicklung und Produktion von verschiedenen Impfstoffen ausgegeben. Diese bis dato einmalige Aktion vieler Länder hat es den Pharmaunternehmen ermöglicht, die Produktion der Impfstoffe auf industriellem Niveau zu starten, obwohl Ergebnisse zur Effizienz und Sicherheit der Impfungen noch nicht vorliegen. Nicht alle Impfstoffe werden zur Produktreife gelangen. Jedoch sind die staatlichen Ausgaben für eventuell unnötige (da nicht anwendbare) Impfstoffe um ein Vielfaches geringer als die Kosten einer verlängerten COVID-19-Pandemie. Bis jetzt liegen (vorläufige) Ergebnisse von drei Impfstoffkandidaten vor.

Es zeigt sich, dass in den vergangenen Monaten große Erfolge bei der Impfstoffentwicklung verbucht werden konnten, und dass somit die Frage nach der Priorisierung derjenigen, die zuerst geimpft werden sollten, in den Vordergrund rückt. Allgemein wird angenommen, dass zunächst Bewohner*innen und Beschäftigte in Pflegeeinrichtungen sowie medizinisches Personal in Krankenhäusern und Arztpraxen geimpft werden. Des Weiteren werden Menschen mit erhöhtem Risiko Vorrang gegenüber jüngeren und weniger morbiden Menschen erhalten. Die finale und detaillierte Empfehlung der ständigen Impfkommission wird für Ende 2020 erwartet.

© Der/die Autor(en) 2020. Open Access: Dieser Artikel wird unter der Creative Commons Namensnennung 4.0 International Lizenz (https://creativecommons.org/licenses/by/4.0/deed.de) veröffentlicht.

Open Access wird durch die ZBW – Leibniz-Informationszentrum Wirtschaft gefördert.

Allerdings ist noch gar nicht ausgemacht, dass Ungleichheiten im Zugang zur Impfung wirklich das größte gesellschaftliche Problem sein werden. Womöglich könnte auch das Gegenteil eintreten: eine zu geringe Impfbereitschaft, um Herdenimmunität zu erreichen. Schon jetzt ist abzusehen, dass nicht jeder, der eventuell von Beginn an impfberechtigt ist, sich auch impfen lassen wird. In Befragungen des Hamburg Center for Health Economics zeigt sich, dass seit Beginn der Pandemie die Impfbereitschaft in Deutschland kontinuierlich gesunken ist und sich mittlerweile bei 57 % stabilisiert hat.

Nun könnte man annehmen, dass die Impfbereitschaft mit dem Risiko eines schweren Verlaufs von COVID-19 zusammenhängt und dass die Impfbereitschaft in den Risikogruppen deutlich höher sein wird. Mit dieser Frage haben sich die Autoren dieses Kommentars beschäftigt und sich das tatsächliche Impfverhalten gegen die Grippe angesehen. In unserer Studie zeigen wir, dass für die Impfneigung die Prudence, also Vorsicht, ausschlaggebend ist. Menschen mit dieser Eigenschaft tendieren dazu, das schlechteste Ergebnis einer Entscheidung zu vermeiden. Bei Impfungen wäre dies die Situation, in der man sich impfen lässt, jedoch trotz Impfung erkrankt. Zudem könnte die Impfung mit Nebenwirkungen, Langzeitfolgen oder anderen Unannehmlichkeiten verbunden sein.

Unsere Untersuchung zeigt: Rund drei Viertel der Befragten kann man als „vorsichtig“ charakterisieren. Entscheidend für die aktuelle Situation ist vor allem das Ergebnis, dass sich „vorsichtige“ Menschen unter ansonsten gleichen Bedingungen seltener gegen die Grippe impfen lassen. Dieser Zusammenhang ist fast ausschließlich in den Risikogruppen, also in der älteren Bevölkerung, sowie bei Menschen mit Vorerkrankungen zu beobachten. Von ihnen ließen sich 68 % der „nicht vorsichtigen“ Menschen tatsächlich impfen, während es unter den „Vorsichtigen“ nur 43 % waren. Gerade bei gefährdeten Menschen, bei denen die Impfung eindeutig empfohlen ist, sorgt die „Vorsicht“ also nicht dafür, dass sie sich impfen lassen, sondern hält sie eher davon ab. Daraus lassen sich Rückschlüsse auf die Bekämpfung der Corona-Pandemie ziehen, zumal sich die Risikogruppen ähneln. Für den Erfolg der Impfung dürfte es entscheidend sein, Sorgen über die Sicherheit und Wirkung eines Impfstoffs ernst zu nehmen und zu adressieren. Studien deuten darauf hin, dass „vorsichtige“ Menschen im Durchschnitt ein höheres Bildungsniveau aufweisen. Die Informationskampagnen sollten daher insbesondere auch Kanäle nutzen, die höher gebildete Menschen erreichen.

Thomas Mayrhofer, Hendrik Schmitz
Hochschule Stralsund und Universität Paderborn
thomas.mayrhofer@hochschule-stralsund.de
